# Risk factors of linezolid-associated hyponatremia from a retrospective case–control study: decreased serum albumin as a selective predictor in elder patients

**DOI:** 10.1080/07853890.2025.2551822

**Published:** 2025-08-31

**Authors:** Liang Liang, Bo-Lin Zhu, Di Chen, Ming Zhao, Yuan-Chao Zhu, Fei Zhao, Peng-Fei Jin, Yang Ju

**Affiliations:** a Department of Pharmacy, Beijing Hospital, National Center of Gerontology, Institute of Geriatric Medicine, Chinese Academy of Medical Sciences, Beijing Key Laboratory of Assessment of Clinical Drugs Risk and Individual Application, (Beijing Hospital), Beijing, China; b Department of Pulmonary and Critical Care Medicine, Beijing Hospital, National Center of Gerontology, Institute of Geriatric Medicine, Chinese Academy of Medical Science, Beijing, China

**Keywords:** Hypoalbuminemia, hyponatremia, linezolid, older patient, risk factors

## Abstract

**Background:**

The incidence of linezolid-associated hyponatremia is approximately 20% in real-world studies. However, there are limited case reports and rare research on linezolid-induced hyponatremia. This study aims to identify risk factors associated with linezolid-induced hyponatremia, particularly in older patients.

**Methods:**

This retrospective study was conducted from January 2018 to December 2023. Patients aged over 18 years who received linezolid for at least three days at Beijing Hospital were included. Statistical analyses were performed using SPSS Statistics version 19.

**Results:**

68 patients were identified in the hyponatremia group, with 68 matched individuals by sex and age in the control group. Binary logistic regression analysis revealed that a history of cardiovascular disease, estimated glomerular filtration rate, and the duration of linezolid therapy were risk factors for hyponatremia. Subgroup analysis in patients age ≥ 65 identified the duration of therapy, a history of cardiovascular disease, a history of endocrine diseases, and serum albumin (ALB) decreased during linezolid therapy as key indicators of hyponatremia.

**Conclusion:**

A decrease in ALB during linezolid therapy is a more selective predictor of hyponatremia than baseline ALB. Linezolid-induced hyponatremia is associated with adverse outcomes, highlighting the importance of monitoring serum sodium and ALB in patients receiving this therapy.

## 1. Background

Albumin is the major circulating protein in human plasma, accounting for up to 60% of plasma proteins [1]. Hypoalbuminemia is very common in critically ill patients, with reported incidences as high as 40–50% [2]. The pharmacokinetics of many drugs changed significantly because of drug-albumin binding. The interaction between the drug and albumin is dynamic equilibrium. In patients with hypoalbuminemia, the reduction in available binding sites leads to an increase in the unbound proportion of drugs [3]. This, in turn, affects the pharmacokinetic and pharmacodynamic factors of highly albumin-bound drugs, such as elevating the concentration of free drug and consequently increasing the risk of toxicity. Additionally, hypoalbuminemia is associated with increased risks of primary and secondary infections [4]. Hypoalbuminemia is a prognostic indicator of poor prognosis in a series of diseases [5–8]. It is also a recognized risk factor for toxicity [9].

Linezolid is an oxazolidinone antibiotics targeted to Gram-positive bacterial including methicillin-resistant Staphylococcus aureus (MRSA), vancomycin-resistant Enterococcus (VRE), and rifampicin-resistant Mycobacterium tuberculosis [10–12]. The oral bioavailability of linezolid is approximately 100%. Linezolid penetrates in various tissue, including the lungs and soft tissue. Linezolid was primarily metabolized through the liver. The manufacturer’s instructions suggest no dosage adjusted in either hepatic or renal insufficiency. Thus, linezolid is widely used in older patients as empirical therapy. However, it is reported that renal insufficiency is a risk factor of adverse effect, such as hematologic toxicity [13] and lactic acidosis [14].

Thrombocytopenia is the most frequently observed side effect of linezolid [15]. The incidence of thrombocytopenia is up to 15–50% in post-marketing studies [16]. Notably, the incidence appears to be higher in older patients. Earlier studies showed a number of risk factors, including linezolid medication duration and trough concentration, age over 75, baseline platelet count and renal dysfunction [17,18]. Hyponatremia is the most common electrolyte disorder. It is an independent risk factor of mortality and length of hospital stay [19]. In a phase III clinical trial, linezolid-induced hyponatremia was noted in 7% of patients. The incidence of hyponatremia is approximately 20% in real-world study [20]. There are only a few case reports and limited research on linezolid-induced hyponatremia [21,22]. Linezolid exposure, C reactive protein (CRP) level before treatment, serum albumin and co-administered potassium-sparing diuretics were identified as potential risk factors [20,21,23].

Linezolid is moderately bound to albumin. The plasma protein binding rate of linezolid is approximately 31%. Prior studies have established a correlation between the plasma level of free linezolid, renal dysfunction and hypoalbuminemia [23]. However, the relationship between hypoalbuminemia and linezolid-associated hyponatremia is still unclear. Therefore, the objectives of this study were to explore (a) the risk factors of linezolid-associated hyponatremia in Chinese patients and (b) the effect of hypoproteinemia on hyponatremia, particularly in older Chinese patients.

## 2. Patients and methods

### 2.1. Ethics

This study received ethical approval from the Ethics Committee of Beijing Hospital (Permit Number: 2023BJYYEC-237-01). Patient consent was waived by the Ethics Committee of Beijing Hospital. As a retrospective study, no interventions were performed on patients while no individual data was published. The study was conducted in accordance with the Declaration of Helsinki and its amendments.

### 2.2. Subjects

This single center retrospective case–control study was conducted between January 2018 and December 2023. Patients over 18 years old who received intravenous and/or oral linezolid (LZD) at a dose of 600 mg every 12 h for at least 3 days in Beijing Hospital were included. Individuals with un-finished laboratory test results and a verified diagnosis of the syndrome of inappropriate antidiuretic hormone secretion (SIADH) or pituitary diseases, pre-treatment sodium level below 130 mEq/L, received other medicines which might induced hypoalbuminemia (e.g. voriconazole, antidepressant, compound sulfamethoxazole) were excluded.

### 2.3. Definition of hyponatremia and hypoalbuminemia

The normal reference value of serum albumin in healthy subjects is 40 g/L ± 10% [3]. Hypoalbuminemia was defined as serum albumin concentration <25 g/L according to the Saline versus Albumin Fluid Evaluation (SAFE) study [2]. In our study, hypoalbuminemia was defined as a serum concentration <3.0 g/dL, while severe hypoalbuminemia was defined as a serum albumin concentration <2.5 g/dL.

Hyponatremia is commonly defined as a serum sodium concentration less than 135 mmol/L, while severe is serum sodium concentration less than 125 mmol/L [24]. In our research, hyponatremia was defined as: (1) sodium level less than 135 mmol/L and a decrease of more than 5% from the baseline or, (2) a nadir serum sodium level less than 130 mmol/L with a decrease of more than 3% from baseline during LZD therapy, while severe hyponatremia was defined as sodium level less than 125 mmol/L or a decrease of more than 10% from the baseline.

### 2.4. Data collection

The following information of patient was collected from their medical records: (1) demographic details (age, gender, comorbidities), (2) primary underlying disease, (3) dosage, frequency and duration of therapy, (4) time of adverse event occurrence, patients outcomes, and 30-day all-cause mortality, (5) clinical laboratory test results, including serum sodium, serum albumin (ALB), renal function [creatinine, blood urea nitrogen (BUN)], liver function [alanine aminotransferase (ALT), aspartate aminotransferase (AST), total bilirubin (TBIL) and direct bilirubin (DBIL)], Platelet (PLT) count, C-reactive protein (CRP), and procalcitonin (PCT).

### 2.5. Statistical analysis

Statistical analyses were performed using SPSS Statistics version 19 (SPSS Inc., Chicago, IL, USA). Categorical variables are presented as numbers (%). In a normal distribution, continuous variables are expressed as mean ± standard deviation (SD). In a non-normal distribution, continuous variables are expressed as median [interquartile range]. Paired *t* tests were used to access the laboratory test results before and after LZD therapy. Differences between the hyponatremia group and the control group were analyzed using the Mann–Whitney *U* test. To explore the risk factors for hyponatremia, a binary logistic regression analysis was conducted. Statistical significance was defined as a *p* value less than 0.05. All the factors identified in univariate analysis (*p* < 0.2) were included in multivariate analysis.

## 3. Results

### 3.1. Patient characteristics

A total of 1700 patients who received linezolid were obtained from the electronic database. Based on the inclusion criteria described above, 528 patients were excluded. Among the remaining 1172 patients, 68 patients developed hyponatremia were included in the hyponatremia group. The other 1104 patients were included in non-hyponatremia group. 68 patients were randomly paired by age and sex from the non-hyponatremia group into the control group. The characteristics of the final cohort are shown in Table 1. The incidence of hyponatremia was 5.80%.

**Table 1.** Base characteristic of the included patients.

	Hyponatremia	Control	*p* value
N	68	68	
Male/female	40/28	40/28	1
Age	70.79 ± 12.95	70.79 ± 12.95	1
Length of hospital stays	32.76 ± 18.04	25.41 ± 9.86	0.001
Site of Infection			
Respiratory	23	23	1
Bloodstream	4	8	0.227
Skin and soft tissue	9	3	0.070
Intra-abdominal	12	10	0.641
Surgical site	4	12	0.033
Febrile neutropenia	1	3	0.310
Complex	7	4	0.345
Others	8	5	0.382
Underlying disease			
Malignant tumors	31	32	0.863
Cardiovascular disease	26	45	0.001
Endocrine diseases	9	20	0.063
Respiratory disease	5	4	0.73
Neurological disease	8	7	0.784
Autoimmune diseases	2	4	0.404
Others	15	16	0.838
Outcome			
Death	17	9	0.081
30-Day all-cause mortality	19	11	0.098

### 3.2. Outcome of patients with hyponatremia

The length of hospital stays and outcomes of patients in both groups were calculated. The length of hospital stay was longer in the hyponatremia group (32.76 days vs 25.41 days, *p* < 0.01). Patient outcomes were worse in the hyponatremia group. 17 patients died in hyponatremia group compared to 9 in control group (*p* = 0.081). A similar result was observed in 30-day all-cause mortality. Compared with the control group, 30-day all-cause mortality was higher in hyponatremia group (27.9% vs 16.2%, *p* = 0.098).

**Figure 1.**
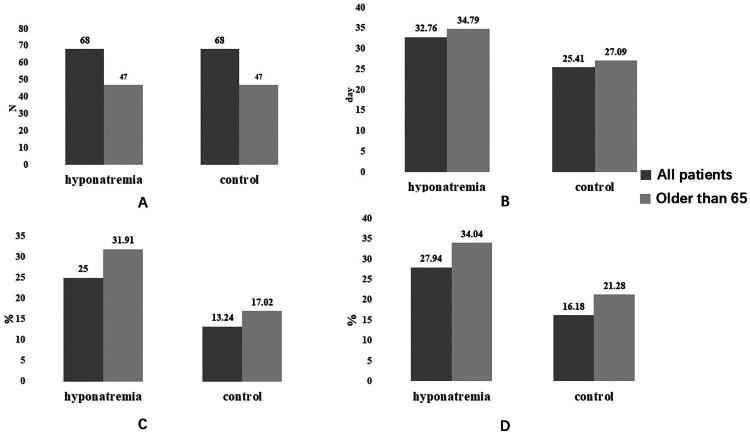
Outcomes of patients who received linezolid (LZD) therapy. A total number of patients; B length of hospital stay; C mortality; D 30-days all-cause mortality.

Similar results were observed in patients older than 65 years. Patients in hyponatremia group displaced longer length of hospital stay than the control group (*p* = 0.003). Both mortality and 30-day all-cause mortality is higher in hyponatremia group (31.91% vs 17.02%, *p* = 0.093; 34.04% vs 21.28%, *p* = 0.167). These results are shown in Figure 1.

### 3.3. Risk factors for hyponatremia

Univariate analysis was used to identify risk factors for hyponatremia. The results are shown in Table 2. The duration of therapy was longer in hyponatremia group than in control group (12 days vs 7 days, *p* < 0.05). Additionally, underlying disease and site of infection differed between the hyponatremia group and the control group.

**Table 2.** Univariate analysis of risk factors.

	Hyponatremia	Control	*p* value
Age ≥65	47	47	1
Duration of therapy	12 (8, 15.75)	7 (5, 11.75)	0.04
Serum sodium	140.47 ± 5.68	140.38 ± 3.87	0.810
AST	24.5 (15.25, 36)	20(14, 33.75)	0.214
ALT	18 (10.25, 38)	17.5(10,28.75)	0.557
TBIL	12.5 (7.6, 17.92)	10.15(7.3, 16.2)	0.345
DBIL > 7	31	24	0.221
ALB	30.99 ± 4.29	31.34 ± 4.22	0.629
ALB change (subtraction of before from after)	0 (–3, 4)	2 (–2, 4)	0.175
ALB decrease	29	19	0.073
CRE	77 (50.25, 123.5)	63 (91, 212)	0.027
BUN	6.635 (4.96, 10.42)	7.16(4.16, 12.82)	0.621
eGFR	87.07 (48.53, 136.20)	68.22 (29.47, 93.13)	0.005
PLT	175(101.5, 265.75)	186(120.25, 247.5)	0.561

AST, aspartate aminotransferase; ALT, alanine aminotransferase; TBIL, total bilirubin; DBIL, direct bilirubin; ALB, serum albumin; CRE, creatinine; BUN, blood urea nitrogen; eGFR, estimated glomerular filtration rate; PLT, platelet.

Considering the relationships between several factors, e.g. TBIL and DBIL, DBIL was selected. Moreover, underlying diseases (cardiovascular disease, endocrine diseases), site of infection (surgical site, skin and soft tissue), duration of linezolid therapy, and eGFR were identified (*p* < 0.2). ALB, PLT and sex were also included in the multivariate analysis. As is shown in Table 3, duration of LZD therapy, previous cardiovascular disease, and eGFR were identified as independent factors for hyponatremia.

**Table 3.** Multivariate analysis of risk factors.

Variables	Wald	*p* value
Cardiovascular disease	4.417	0.036
Duration of therapy	12.157	<0.001
eGFR	3.074	0.008

eGFR, estimated glomerular filtration rate.

### 3.4. Relationship between hypoalbuminemia and hyponatremia

In both univariate analysis and multivariate analysis, ALB level was not related to hyponatremia. However, in multivariate analysis, a decrease of serum albumin during LZD therapy was an independent indicator of hyponatremia (Table 4). In other words, hyponatremia was more likely to occur in patients who developed hypoalbuminemia during LZD therapy.

**Table 4.** Adjusted risk factors.

Variables	Wald	*p* value
Cardiovascular disease	3.559	0.059
Duration of therapy	13.526	<0.001
eGFR	2.82	0.093
ALB decrease	3.627	0.057

eGFR, estimated glomerular filtration rate; ALB, serum albumin.

### 3.5. Sub-group analysis

Univariate and multivariate analyses were carried out in patients older than 65 years. The results are shown in Table 5. Duration of therapy, eGFR, serum albumin decreases during LZD therapy, previous cardiovascular disease and previous endocrine diseases were identified as indicators of hyponatremia.

**Table 5.** Univariate and multivariate analysis in patients older than 65 years.

Variables	Univariate analysis	Multivariate analysis
Duration of therapy	<0.001	0.01
Cardiovascular disease	<0.001	0.021
Endocrine Diseases	0.002	0.026
Surgical site	0.025	–
eGFR	0.035	–
ALB decrease	0.034	0.018
DBIL >7	0.207	–

eGFR, estimated glomerular filtration rate; ALB, serum albumin; DBIL, direct bilirubin.

## 4. Discussion

Hyponatremia is the most common electrolyte imbalance in clinical practice and can lead to a range of clinical symptoms, from subtle to severe [25]. Many medications can induce hyponatremia, including arginine vasopressin analogs, anticancer chemotherapeutic agents, psychotropic agents, antipsychotics, antidepressants, anticonvulsants, and thiazide diuretics [26].

Instances of antibiotic-induced hyponatremia have been documented in case reports, including those related to trimethoprim-sulfamethoxazole [27], voriconazole [28], and ciprofloxacin [29]. In real-world studies, the incidence of hyponatremia is approximately 20% [20]. In clinical trials, the incidence of hyponatremia is approximately 7%. However, in our study, the observed incidence of hyponatremia was lower. This could be attributed to the strict definition of hyponatremia employed in our research. In our study, hyponatremia was defined as a sodium level <135 mmol/L with a decrease of more than 5% from baseline, or a sodium level <130 mmol/L with a decrease of more than 3%. Consequently, patients with only a slight reduction in sodium levels were excluded from the hyponatremia group. Additionally, based on the exclusion criteria, patients co-administered with drugs known to cause hyponatremia (such as voriconazole), were also excluded. These factors might explain the lower incidence of hyponatremia in our study.

Hyponatremia is an established independent risk factor for mortality and length of hospital stay. Our study is the first to specifically describe the relationship between LZD-induced hyponatremia and outcome. Compared to the control group, patients with hyponatremia exhibited longer hospital stays. Notably, patients who developed hyponatremia during LZD therapy had a higher risk of mortality both in the overall patient population and specifically in those aged ≥65 years. Therefore, identifying the risk factors associated with LZD-induced hyponatremia is of utmost importance.

Take consideration that elderly women are at higher risk for hyponatremia, the patients in control group were matched by age and sex. Therefore, age was not included in the final analysis. Through multivariate analysis, it was determined that eGFR was linked to an elevated risk of hyponatremia. eGFR was identified as the risk factor for LZD-associated thrombocytopenia [30]. Nearly 35% of linezolid was recovered in urine in its original form, while 50% was eliminated through extrarenal clearance as morpholine ring-oxidized inactive metabolites. In patients with severe renal failure, neither plasma concentration of linezolid nor area under the plasma concentration/time curve increased significantly [31]. Clearance of linezolid decreases in some pathological situations, such as severe liver failure (approximately 50%) [32] and end-stage renal failure (approximately 20%) [33]. It is reported that linezolid exposure is an independent factor contributing to hyponatremia [34].

Previous cardiovascular disease, endocrine diseases and duration of LZD therapy were also recognized as risk factors. Consistent with previous reports, prolonged duration of LZD therapy was associated with an increased risk of hyponatremia [21,35]. Hyponatremia cut-off values for LZD therapy duration were 9.5 days in Yang’s report while 14 days in Tanaka’s report. Incidence of hyponatremia is positive correlated to duration of LZD.

ALB was not selected as a risk factor in either univariate analysis or multivariate analysis. However, an interesting phenomenon was observed. Considering ALB change during LZD therapy, a significant positive correlation was detected between the decrease in serum albumin during LZD therapy and hyponatremia. In contrast to *Tanaka’s* report [21], hypoalbuminemia that occurred during LZD therapy, rather than prior to it, was more likely to serve as an indicator of hyponatremia. Therefore, it is essential to monitor the ALB level during LZD therapy. In patients with lower ALB level before LZD therapy, prompt supplementation with human serum albumin to correct hypoalbuminemia may potentially reduce the incidence of hyponatremia.

The primary contributing factor for drug-induced hyponatremia is syndrome of inappropriate antidiuresis (SIAD) [26]. SIAD can be further classified into the syndrome of inappropriate antidiuretic hormone secretion (SIADH) and nephrogenic syndrome of inappropriate antidiuresis (NSIAD). In several case reports, SIADH was considered as the predominant factor of LDZ induced hyponatremia [22,36]. LZD is a reversible, nonselective inhibitor of monoamine oxidase. It has been proposed that the effects of norepinephrine on α1-adrenergic receptors and the effect of serotonin on 5- hydroxy tryptamine receptors might contribute to SIADH [37]. Direct stimulation of AVP release in CNS might also be a mechanism of hyponatremia [36]. The plasma protein binding of linezolid is approximately 31%. Free linezolid plasma concentration is higher in hypoalbuminemia patients compared to those without hypoalbuminemia [23]. Overexposure to linezolid is highly prevalent when serum albumin decreases during LZD therapy, resulting in higher concentrations in the central nervous system.

Another hypothesis considered hyponatremia in the context of hypoalbuminemia is that LZD stimulates the central ADH secretory pathway and elevates ADH levels [21]. Under the condition of hypoalbuminemia, decreased oncotic pressure leads to reduced effective plasma volume. Baroreceptors detected decreased pressure. The renin–angiotensin–aldosterone system is then activated. The ADH secretion system responds in a compensatory manner. The decrease in ALB may amplify the uptake process. Consequently, ALB decrease during LZD therapy predicts hyponatremia more accurately than ALB before LZD therapy.

Nevertheless, this study had several limitations. LZD exposure was not evaluated in our research. Therefore, the relationship between LZD expose and hyponatremia was not accessed directly. Tanaka et al. demonstrated an association between the C-reactive protein level before LZD and hyponatremia [20]. However, due to missing data, CRP was not included in our final analysis. The mechanism underlying LZD-induced hyponatremia remains elusive. Risk factors identified from different studies were heterogeneous. Hence, further studies are needed to explore the mechanism and risk factors of LZD-induced hyponatremia.

## Data Availability

Data are available upon request to the corresponding author.
